# Dietary bile acid supplementation in weaned piglets with intrauterine growth retardation improves colonic microbiota, metabolic activity, and epithelial function

**DOI:** 10.1186/s40104-023-00897-2

**Published:** 2023-07-13

**Authors:** Yang Liu, Md. Abul Kalam Azad, Sujuan Ding, Qian Zhu, Francois Blachier, Zugong Yu, Haijun Gao, Xiangfeng Kong

**Affiliations:** 1 Key Laboratory of Agro-Ecological Process in Subtropical Region, Hunan Provincial Key Laboratory of Animal Nutritional Physiology and Metabolic Process, Institute of Subtropical Agriculture, Chinese Academy of Sciences, Changsha, 410125 Hunan China; 2 College of Veterinary Medicine, Nanjing Agricultural University, Nanjing, Jiangsu 210095 China; 3 Université Paris-SaclayAgroParisTech, INRAE, UMR PNCA, 75005, Paris, France; 4 College of Medicine, Howard University, Washington, DC, 20059 USA

**Keywords:** Bile acid, Intrauterine growth retardation, Metabolite, Microbiota, Piglet

## Abstract

**Background:**

Intrauterine growth retardation (IUGR) is one of the major constraints in animal production. Our previous study showed that piglets with IUGR are associated with abnormal bile acid (BA) metabolism. This study explored whether dietary BA supplementation could improve growth performance and colonic development, function, microbiota, and metabolites in the normal birth weight (NBW) and IUGR piglets. A total of 48 weaned piglets (24 IUGR and 24 NBW) were allocated to four groups (12 piglets per group): (i) NBW group, (ii) NBW + BA group, (iii) IUGR group, and (iv) IUGR + BA group. Samples were collected after 28 days of feeding.

**Results:**

The results showed that dietary BA supplementation increased the length and weight of the colon and colon weight to body weight ratio, while decreased the plasma diamine oxidase (DAO) concentration in the NBW piglets (*P* < 0.05). Dietary BA supplementation to IUGR piglets decreased (*P* < 0.05) the plasma concentrations of *D*-lactate and endotoxin and colonic DAO and endotoxin, suggesting a beneficial effect on epithelial integrity. Moreover, dietary BA supplementation to NBW and IUGR piglets increased Firmicutes abundance and decreased Bacteroidetes abundance (*P* < 0.05), whereas *Lactobacillus* was the dominant genus in the colon. Metabolome analysis revealed 65 and 51 differential metabolites in the colon of piglets fed a diet with/without BA, respectively, which was associated with the colonic function of IUGR piglets. Furthermore, dietary BA supplementation to IUGR piglets upregulated the expressions of *CAT*, *GPX*, *SOD*, *Nrf1*, *IL-2*, and *IFN-γ* in colonic mucosa (*P* < 0.05).

**Conclusions:**

Collectively, dietary BA supplementation could improve the colonic function of IUGR piglets, which was associated with increasing proportions of potentially beneficial bacteria and metabolites. Furthermore, BA shows a promising application prospect in improving the intestinal ecosystem and health of animals.

**Supplementary Information:**

The online version contains supplementary material available at 10.1186/s40104-023-00897-2.

## Background

Intrauterine growth retardation (IUGR) is very common in animal production, and several studies have revealed its underlying mechanisms [1]. The incidence of IUGR is approximately 15%–20% in neonatal piglets [2], and IUGR affects pig health and production. For example, a previous study indicated that IUGR generally results in high neonatal mortality, gut dysfunction, low nutrient utilization efficiency, and growth limitation after birth [3]. A previous study reported that IUGR impairs intestinal development in piglets by altering their intestinal structure and transcriptomic expression of proteins [4]. Furthermore, impaired antioxidant capacity caused by IUGR results in oxidative stress injury in weaned piglets [5]. For example, IUGR causes intestinal mucosal injury by increasing the abundance of proteins associated with oxidative stress and reducing the antioxidant capacity [6]. In addition, the intestinal microbiota can accelerate the maturation of innate immunity and the barrier function of the intestine and host health [7]. Piglets with IUGR are characterized by lower intestinal bacterial diversity and taxonomic abundance, and the effects of IUGR on intestinal metabolite production have been described previously [2]. Numerous metabolites produced by intestinal bacteria are essential for intestinal epithelial metabolism and function [8] and the peripheral tissues of mammals after absorption and metabolism [9]. The altered plasma metabolites in IUGR piglets are mainly related to fatty acid metabolism and inflammatory responses in weaned piglets [10].

Primary bile acids (BAs) are mainly synthesized in hepatocytes and play important roles in intestinal nutrient absorption, energy homeostasis, toxic metabolite production, and xenobiotic metabolism [11]. Secondary BAs are important intestinal metabolites. However, the role of secondary BAs in the colonic ecosystem remains unclear. Some beneficial effects, such as the capacity of deoxycholic acid to inhibit the colonization of the pathogen *Clostridium difficile* have been reported previously by Buffie et al. [12]. In contrast, excessive deoxycholic acid appears cytotoxic to colonic epithelial cells [13] and alters the colonic intestinal barrier function [14]. Furthermore, BA can modulate microbial abundance in the intestine. Accumulating research evidence suggests that the intestinal microbiota and BA play pivotal roles in intestinal homeostasis and inflammation [15]. Dysbiosis of the intestinal microbiota and BA metabolism can impair intestinal barrier function and immunity, and abnormal BA metabolism is associated with inflammatory bowel diseases [16].

Microbiota harbored in the mammalian colon not only have the major function of fermenting undigested dietary compounds, but also affect the metabolic, developmental, and physiological processes of the host [17]. However, the effects of colonic metabolites (including BA) on colonic health in normal birth weight (NBW) and IUGR piglets are still unclear. Therefore, we hypothesized that dietary BA supplementation might positively affect the colonic microbiota and metabolite production, thus improving the intestinal health of IUGR piglets. Therefore, using 16S rRNA sequence and non-target metabolome technologies, this study explored whether BA supplementation can regulate growth performance (including average daily gain (ADG), average daily feed intake (ADFI), and the ratio of ADG to ADFI (G/F)), diarrhea, colonic microbial community and metabolites, colonic immune, antioxidant, and barrier-related gene expression in IUGR piglets in comparison with the NBW piglets.

## Materials and methods

### Animals and experimental design

Twenty-four pregnant sows (Landrace × Large White) with similar health conditions during 3−5 parities were selected from a herd at Yiyang, Hunan, China. After birth, 48 experimental piglets were selected from 24 litters (one IUGR and one NBW piglet from each litter). Piglets with the highest birth weight and the lowest birth weight in the same litter were selected for the NBW and IUGR groups, respectively [2]. Piglets were weaned at 21 d and transferred to individual pens. After 7 d adaption, 24 NBW piglets (7.43 ± 0.18 kg) and 24 IUGR piglets (5.86 ± 0.20 kg) were randomly assigned to four groups based on  body weight (BW). The experimental groups were as follows: (i) NBW, NBW piglets fed a basal diet; (ii) NBW + BA, NBW piglets fed a basal diet supplemented with 400 g/t BA; (iii) IUGR, IUGR piglets fed a basal diet; and (iv) IUGR + BA, IUGR piglets fed a basal diet supplemented with 400 g/t BA. Dietary BA was obtained from Longchang Animal Health Product Co., Ltd. (Shandong, China). The purity of bile acid was ≥ 98.5%, derived from pig bile consisted chenodeoxycholic acid (17%), hyodeoxycholic acid (68%), and hyocholic acid (9%). All weaned piglets were housed in a controlled temperature (23–25 ºC) and humidity (60% ± 5%) room and had free access to food and drinking water at all times. The piglets were housed in an environmentally controlled facility with hard plastic and slatted flooring. Each pen (0.6 m × 1.2 m) was equipped with a single-hole feeder and a water nipple. Piglets were fed three times per day at 8:00, 13:00, and 18:00 with their respective diets. The composition and nutrient levels of the basal diet (Additional file 1: Table S1) for weaned piglets met the recommendations of the National Research Council [18]. The animal experiments lasted 28 d.

### Sample collection

All piglets were weighed 12 h after the last feeding and euthanized by a jugular puncture after anesthesia on d 28 of the experiment. Blood samples were drawn from each piglet and centrifuged at 3,000 × *g* for 10 min at 4 ºC to obtain plasma to determine diamine oxidase (DAO), *D*-lactate, and endotoxin concentrations. The contents of colon samples were collected from the middle section of the whole colon, immediately frozen in liquid nitrogen, and then stored at −80 ºC until bacterial genomic DNA extraction and determination of short-chain fatty acid (SCFA) concentrations. Colonic mucosa samples were collected from the remaining colonic segments as described previously [19] and stored at −80 ºC for total mRNA extraction.

### Evaluation of piglets’ growth performance

All piglets were weighed on d 0, 14, and 28 of the trial. The ADG, ADFI, G/F, and diarrhea rate were calculated as previously described [20]. The diarrhea rate (%) was calculated as follows: total diarrhea times/(total number of piglets) × trial days × 100.

### Colonic microbiota analysis

The total microbial genomic DNA was extracted from colonic contents (200 mg) using a QIAamp DNA Stool Mini Kit (Germantown, MD, USA), according to the manufacturer’s instructions. The DNA concentration was determined using a NanoDrop instrument (Thermo Fisher Scientific, MA, USA), and the quality was measured using the 1.2% agarose gel electrophoresis. The V3−V4 region of the 16S rRNA gene was amplified using universal primers, including the forward primer 341F (5′-ACTCCTACGGGAGGCAGCA-3′) and reverse primer 806R (5′-TCGGACTACHVGGGTWTCTAAT-3′). Amplicons and 16S rRNA sequencing were performed as previously described [19].

Sequencing analyses of 16S rRNA were based on amplicon sequence variants (ASVs) and were conducted on the QIIME2 (Quantitative Insights into Microbial Ecology, v 2.0) platform (Personal Bio, Shanghai, China). Alpha diversity indices, including Chao 1, Observed species, Shannon index, and Simpson index, were used to determine the evenness of colonic microbiota. Partial least squares discriminant analysis (PLS-DA) and principal coordinate analysis (PCoA) were used to evaluate the beta diversity. Linear discriminant analysis (LDA) combined with effect size measurements (LEfSe) was used to identify biomarkers in the four groups.

### Liquid chromatography-mass spectrometry (LC–MS) analysis

LC–MS analysis was performed by BioNovoGene Co., Ltd. (Suzhou, China). Briefly, approximately 100 mg of colonic content was collected, transferred into a 2-mL Eppendorf tube, and then added 0.6 mL methanol. The mixture was then centrifuged at 12,000 × *g* for 10 min at 4 ºC. The supernatant was filtered for LC–MS analysis using a 0.22-µm membrane. A 20-µL sample was retained for quality control. Raw data were obtained from LC–MS and analyzed using the Proteowizard (v3.0.8789). Positive and negative ion modes from the Q Exactive HFX mass spectrometer (Thermo Fisher Scientific, MA, USA) were used for all samples. There were 6,405 extracted peaks and 3,937 retained peaks after the sample pre-processing. The resulting peaks were imported into soft independent modelling of class analogy (SIMCA) software (V15.0.2). The metabolite annotation of the data was referenced to the METLIN (metlin.scripps.edu) and mzCloud databases (www.mzcloud.org). Unsupervised principal component analysis (PCA) and orthogonal partial least squares discriminant analysis (OPLS-DA) were performed to visualize the separation and identify different metabolites among the groups. The metabolites were considered to be significantly different when VIP > 1 and *P* < 0.05 (Student’s *t*-test).

### Determination of the colonic metabolites

The SCFAs concentrations were determined using gas chromatography (7890A, Agilent, Santa Clara, USA), as described in our previous study [20]. Briefly, approximately 1.00 g of colonic content was mixed with 5 mL of ultrapure water and centrifuged at 1,000 × *g* for 10 min to obtain the supernatant. Then, the supernatant fluid and 25% metaphosphoric acid were mixed (9:1, v/v) in a 2.0-mL centrifuge tube and centrifuged at 2,000 × *g* for 10 min at 4 ºC. Subsequently, the supernatant was filtered through a 0.45-μm polysulfone filter for gas chromatography analysis. The SCFAs included acetate, butyrate, isobutyrate, isovalerate, propionate, and valerate.

### RNA isolation and real-time quantitative PCR analysis

The colonic mucosa sample (100 mg) was used to extract the total RNA using the TRIzol reagent (AG, Changsha, China). The concentration and quality of colonic mucosal total RNAs were determined using a NanoDrop instrument (Thermo Fisher Scientific, MA, USA). The total RNA (1 µg) was reverse-transcribed to cDNA using the commercial RT reagent kit (AG, Changsha, China) and Light Cycler® 480 II RT-PCR System (Roche, Basel, Switzerland), as described previously [19, 21]. Pig-specific primers used in this study are listed in Additional file 2: Table S2. The targeted gene expression was calculated using the 2^−△△Ct^ value [17], and β-actin was used as the internal control.

### Statistical analysis

All data (except colonic microbiota and metabolite data) were analyzed in a 2 × 2 factorial arrangement by ANOVA using the general linear model procedure in SPSS 22.0 (SPSS Inc., Chicago, USA). The statistical model included IUGR, BA, and their interactions. Differences in means in the different groups were performed using the Tukey–Kramer *post-hoc* test when the interaction was valid (*P* < 0.05). The piglets were individually penned in this study, and the experimental pens were used as experimental units. The gene expression data related to colon function were analyzed by two-way ANOVA using the individual piglets as experimental units. *P*-values < 0.05 represent significant differences. The correlations between different colonic microbial genera and metabolites were assessed using Spearman’s correlation test.

## Results

### Effects of BA on the growth performance of weaned piglets with NBW and IUGR

The effects of dietary BA on the growth performance of weaned piglets with NBW and IUGR piglets are presented in Table 1. Compared with the NBW piglets, the IUGR piglets had the higher initial and final BW, while had lower ADG and ADFI during d 1–14 of the trial (*P* < 0.05). The IUGR piglets had the lower (*P* < 0.05) ADG and G/F during d 15−28 of the trial, as well as the ADG and G/F throughout the trial (d 1−28), when compared with the NBW piglets. Furthermore, compared with the NBW piglets, IUGR piglets had the lower (*P* < 0.05) colon length, weight, and the ratio of colon weight to BW, while dietary BA supplementation increased (*P* < 0.05) the colon weight, length, and the ratio of colon weight to BW of the NBW piglets. Furthermore, there were significant interactions (*P* < 0.05) between BA and IUGR on colon weight, length, and the ratio of colon weight to BW of the weaned piglets.

**Table 1:** Effects of dietary bile acid (BA) supplementation on the growth performance of weaned piglets with NBW and IUGR

Items	NBW		IUGR		SEM	*P*-values		
	− BA	** + **BA	− BA	** + **BA		IUGR	BA	IUGR × BA
Initial weight, kg	7.48	7.38	5.91	5.81	0.18	< 0.01	0.72	0.98
Final weight, kg	18.67	17.05	15.06	15.33	0.37	< 0.01	0.29	0.14
1−14 d								
ADG, kg/d	0.36	0.32	0.31	0.30	0.01	0.03	0.19	0.35
ADFI, kg/d	0.68	0.65	0.57	0.61	0.01	< 0.01	0.76	0.10
G/F	0.53	0.48	0.52	0.48	0.03	0.76	0.06	0.94
15−28 d								
ADG, kg/d	0.44	0.39	0.35	0.34	0.01	< 0.01	0.19	0.35
ADFI, kg/d	0.99	0.99	0.99	0.94	0.01	0.40	0.32	0.32
G/F	0.44	0.39	0.34	0.36	0.02	< 0.01	0.59	0.06
1−28 d								
ADG, kg/d	0.40	0.36	0.33	0.32	0.01	< 0.01	0.19	0.35
ADFI, kg/d	0.78	0.77	0.75	0.74	0.01	0.11	0.57	0.86
G/F	0.51	0.46	0.43	0.43	0.02	< 0.01	0.21	0.23
Colon weight, g	170.82^c^	275.55^a^	209.28^b^	207.14^bc^	8.56	0.27	< 0.01	< 0.01
Colon length, cm	106.77^c^	196.08^a^	158.58^b^	167.36^ab^	6.90	0.27	< 0.01	< 0.01
Colon weight/BW	9.19^b^	15.81^a^	14.18^a^	14.04^a^	0.52	0.04	< 0.01	< 0.01
Diarrhea rate, %	4.83	5.08	5.40	6.23	0.01	0.75	0.84	0.91

Values are expressed as means with pooled SEM (*n* = 11 or 12). *P* < 0.05 represents significantly difference. ^a−c^Means with different superscript letters in the same row are significantly different (*P* < 0.05). *ADG* Average daily gain, *ADFI* Average daily feed intake, *G/F* The ratio of ADG to ADFI, *BW* Body weight, *NBW* Normal birth weight, *IUGR* Intrauterine growth retardation, − *BA* A basal diet without BA, + *BA* A basal diet supplemented with 400 g/t BA

### Effects of BA on the colonic barrier function of weaned piglets with NBW and IUGR

As shown in Table 2, compared with the NBW piglets, colonic DAO concentration was increased (*P* < 0.05) in the IUGR piglets with/without BA supplementation. Dietary BA supplementation decreased (*P* < 0.05) the plasma *D*-lactate and DAO concentrations in the IUGR and NBW piglets. Furthermore, there was no interaction between BA and IUGR on the colonic barrier function of weaned piglets (*P* > 0.05).

**Table 2:** Effects of dietary bile acid (BA) supplementation on colonic barrier function and short-chain fatty acids (SCFA) concentrations in weaned piglets with NBW and IUGR

Items	NBW		IUGR		SEM	*P*-values		
	− BA	+ BA	− BA	+ BA		IUGR	BA	IUGR × BA
Plasma								
* D*-lactate, μg/mL	1.29	1.17	1.61	1.11	0.06	0.26	< 0.01	0.11
DAO, IU/L	181.97	138.63	187.34	147.16	7.70	0.63	< 0.01	0.91
Endotoxin, pg/mL	308.22	289.77	362.97	253.37	16.80	0.78	0.06	0.17
Colon								
* D*-lactate, μg/mL prot	0.34	0.31	0.51	0.37	0.03	0.08	0.14	0.34
DAO, IU/L prot	39.91	35.80	60.22	40.52	3.19	0.04	0.05	0.19
Endotoxin, pg/mL prot	308.22	289.77	362.97	253.37	8.08	0.53	0.08	0.05
Colon SCFA concentrations, mg/g								
Acetate	4.20	3.65	4.07	4.07	0.13	0.58	0.30	0.29
Butyrate	1.39	0.89	1.42	0.98	0.06	0.56	< 0.01	0.83
Isobutyrate	0.18	0.19	0.17	0.16	0.01	0.11	0.75	0.40
Valerate	0.26	0.23	0.22	0.17	0.01	0.02	0.11	0.70
Isovalerate	0.30	0.32	0.26	0.26	0.01	0.05	0.67	0.72
Propionate	1.70	1.47	1.74	1.41	0.05	0.92	< 0.01	0.60
Total SCFA	8.02	6.76	7.87	7.04	0.21	0.88	0.01	0.60

Values are expressed as means with pooled SEM (*n* = 11 or 12). *P* < 0.05 represents significant difference. *NBW* Normal birth weight, *IUGR* Intrauterine growth retardation, − *BA* A basal diet without BA, + *BA* A basal diet supplemented with 400 g/t BA

### Effects of BA on the colonic microbial diversity of weaned piglets with NBW and IUGR

The Chao 1 index of IUGR piglets was decreased (*P* < 0.05) compared with the NBW piglets. Dietary BA supplementation decreased (*P* < 0.05) the Chao 1 and Shannon indices in both NBW and IUGR piglets (Additional file 3: Fig. S1). The PCoA analysis showed significant separations between the NBW and NBW + BA groups, as well as the IUGR and IUGR + BA groups. Moreover, PLS-DA also showed significant separations among the different treatment groups (Additional file 4: Fig. S2), which indicates that dietary BA supplementation altered the structural characteristics of the colonic microbiota in the NBW and IUGR piglets and the IUGR piglets had different colonic microbial structural characteristics from the NBW piglets.

### Effects of BA on the colonic microbial community of weaned piglets with NBW and IUGR

**Fig. 1:**
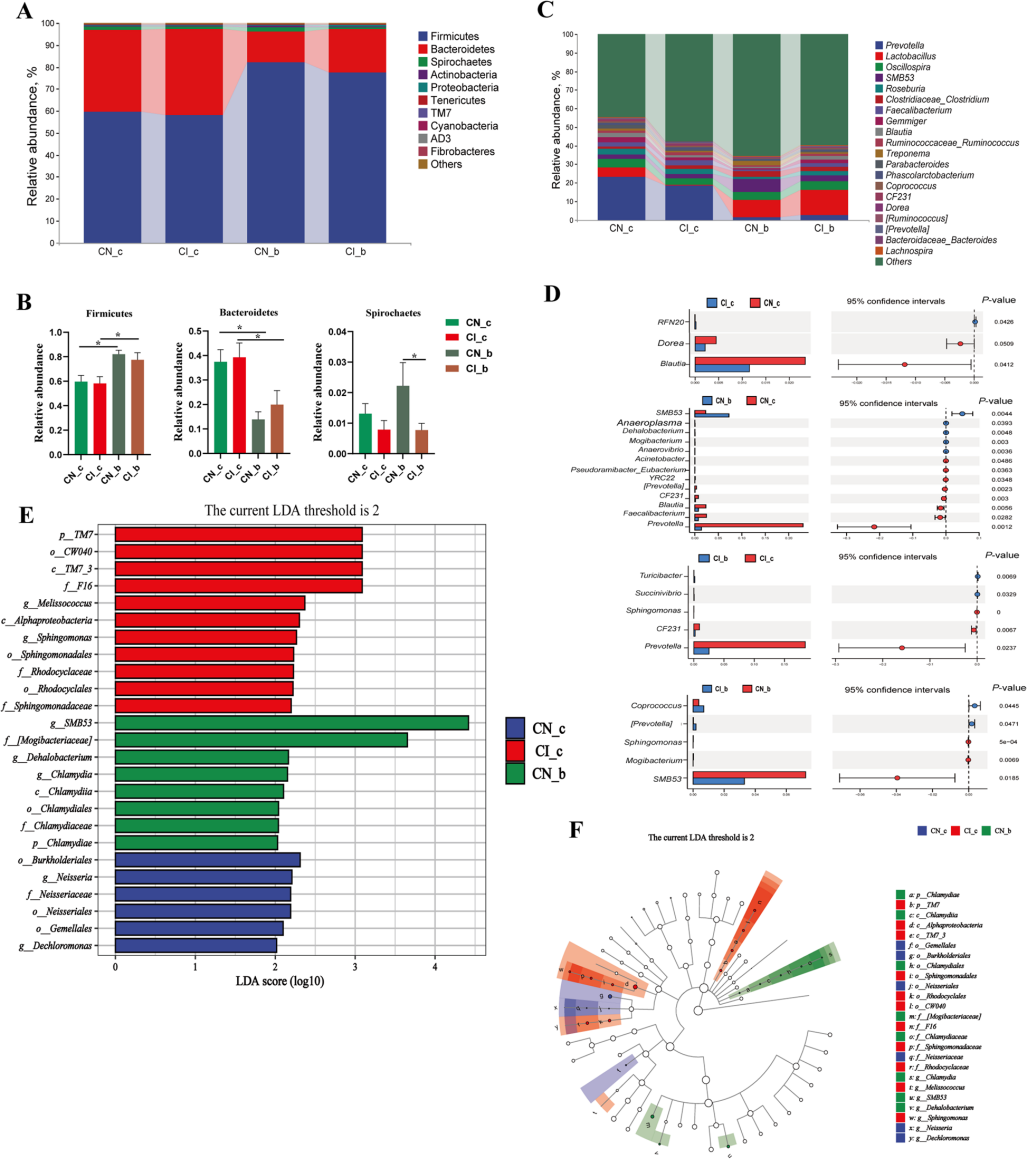
Effects of dietary bile acid (BA) supplementation on the composition and differences in colonic microbial community in weaned piglets with normal birth weight (NBW) and intrauterine growth retardation (IUGR) at the phylum (**A** and **B**) and genus (**C** and **D**) levels. **E** represents the histogram of LDA value distribution; **F** represents the species taxonomy cladogram. The color nodes represent the microbial species with a significant difference, while white nodes indicate no significant difference (*n* = 11 or 12). ^*^P < 0.05. *N_c* NBW group (NBW piglets fed a basal diet), *I_c* IUGR group (IUGR piglets fed a basal diet), *N_b* NBW + BA group (NBW piglets fed a basal diet supplemented with 400 g/t BA), *I_b* IUGR + BA group (IUGR piglets fed a basal diet supplemented with 400 g/t BA)

The top four abundant phyla in the NBW group were Firmicutes (59.62%), Bacteroidetes (37.44%), Proteobacteria (0.35%), and Spirochaetes (1.31%), whereas in the IUGR group were Firmicutes (58.19%), Bacteroidetes (39.28%), Spirochaetes (0.79%), and Actinobacteria (0.49%); the top four abundant phyla in the NBW + BA group were Firmicutes (82.10%), Bacteroidetes (13.91%), Spirochaetes (2.22%), and Actinobacteria (0.43%), whereas in the IUGR + BA group, Firmicutes (77.46%), Bacteroidetes (19.93%), Spirochaetes (0.77%), and Proteobacteria (0.57%) were the most abundant (Fig. 1A). Furthermore, dietary BA supplementation to weaned piglets increased Firmicutes abundance and decreased Bacteroidetes abundance regardless of IUGR status (*P* < 0.05). Moreover, dietary BA supplementation to IUGR piglets decreased (*P* < 0.05) Spirochaetes abundance compared to the NBW piglets supplemented with BA (Fig. 1B).

*Prevotella* (23.05%), *Lactobacillus* (5.09%), *Oscillospira* (4.87%), and *Roseburia* (3.07%) were the most abundant genera in the NBW group, whereas *Prevotella* (18.44%), *Oscillospira* (3.61%), *Faecalibacterium* (2.85%), and *Roseburia* (2.72%) were the most abundant genera in the IUGR group. *Lactobacillus* (9.23%), *SMB53* (7.25%), *Oscillospira* (4.23%), and *Clostridium* (2.99%) were the most abundant in the NBW + BA group, whereas *Lactobacillus* (13.76%), *Oscillospira* (4.41%), *SMB53* (3.30%), and *Clostridium* (2.55%) were the most abundant genera in the IUGR + BA group (Fig. 1C). IUGR piglets had a higher abundance of *RNF20* but lower abundances of *Dorea* and *Blautia* compared with the NBW group (*P* < 0.05). The relative abundances of *SMB53*, *Anaeroplasma*, *Dehalobacterium*, *Mogibacterium*, and *Anaerovibrio* were higher (*P* < 0.05), but *Acinetobacter*, *Eubacterium*, *YRC22*, [*Prevotella*], *CF231*, *Blautia*, *Faecalibacterium*, and *Prevotella* were lower (*P* < 0.05) in the NBW + BA group compared with the NBW group. In addition, the relative abundances of *Turicibacter* and *Succinivibrio* were higher (*P* < 0.05), whereas *Sphingomonas*, *CF231*, and *Prevotella* were lower (*P* < 0.05) in the IUGR + BA group compared with the IUGR group. Furthermore, the relative abundances of *Coprococcus* and *Prevotella* were higher (*P* < 0.05) but *Sphingomonas*, *Mogibacterium*, and *SMB53* were lower (*P* < 0.05) in the IUGR + BA group compared with the NBW + BA group (Fig. 1D).

### Effects of BA on the colonic microbial function of weaned piglets with NBW and IUGR

The effects of BA supplementation on the colonic microbial function of weaned piglets are shown in Fig. 1E. Six colonic microbiota biomarkers were enriched in the NBW group: Burkholderiales, *Neisseria*, Neisseriaceae, Neisseriales, Gemellales, and *Dechloromonas*. Eleven colonic microbiota biomarkers were enriched in the IUGR group: p_TM7, o_CW040, c_TM7-3, f_F16, *Melissococcus*, Alpha_Proteobacteria, *Sphingomonas*, Sphingomonadales, Rhodocyclaceae, Rhodocyclales, and Sphingomonadaceae. Moreover, g_*SMB53*, [Mogibacteriaceae], *Dehalobacterium*, *Chlamydia*, Chlamydiia, Chlamydiales, Chlamydiaceae, and Chlamydiae were enriched in the NBW + BA group. However, dietary BA supplementation to IUGR piglets did not affect colonic microbial biomarker enrichment (Fig. 1E). Chlamydiae was the most dominant microbiota in the NBW + BA group, *Gemellales* was the most dominant microbiota in the NBW group, and p_TM7 and Alpha_Proteobacteria were the most dominant in the IUGR group (Fig. 1F).

**Fig. 2:**
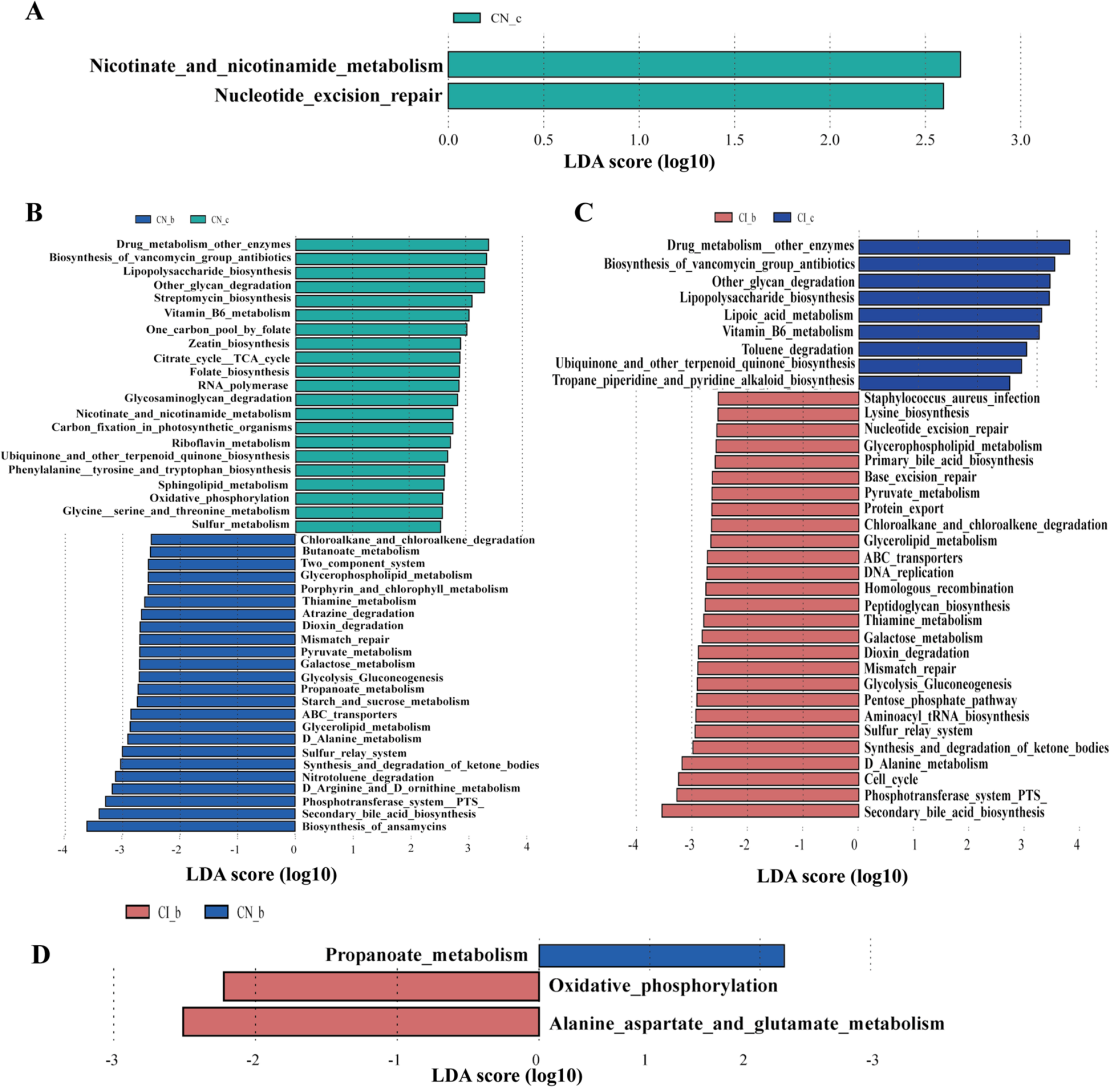
Predictive metagenomics showing differences in the function of colonic microbiota in weaned piglets with normal birth weight (NBW) and intrauterine growth retardation (IUGR) using PICRUSt analysis at level 3 (*n* = 11 or 12). **A** represents N_c vs. I_c groups; **B** represents N_c vs. N_b groups; **C** represents I_c vs. I_b groups; **D** represents N_b vs. I_b groups. *N_c* NBW group (NBW piglets fed a basal diet), *I_c* IUGR group (IUGR piglets fed a basal diet), *N_b* NBW + BA group (NBW piglets fed a basal diet supplemented with 400 g/t BA), *I_b* IUGR + BA group (IUGR piglets fed a basal diet supplemented with 400 g/t BA)

As shown in Fig. 2, IUGR piglets had lower (LDA > 2.5, *P* < 0.05) abundances of microbial genes associated with nicotinate/nicotinamide metabolism and nucleotide excision repair compared to those in the NBW piglets (Fig. 2A). Dietary BA supplementation to NBW piglets increased (LDA > 3, *P* < 0.05) the relative abundances of microbial genes involved in the nitrotoluene degradation, *D*-arginine/*D*-ornithine metabolism, phosphotransferase system, and secondary BA and ansamycins biosynthesis. In contrast, BA supplementation decreased (LDA > 2.5, *P* < 0.05) the relative abundances of microbial genes involved in drug metabolism by other enzymes, biosynthesis of vancomycin group antibiotics, lipopolysaccharide biosynthesis, glycan degradation, streptomycin biosynthesis, vitamin B_6_ metabolism, and one-carbon pool by folate in the NBW piglets (Fig. 2B).

Dietary BA supplementation to IUGR piglets decreased (LDA > 3, *P* < 0.05) the relative abundances of microbial genes involved in drug metabolism by other enzymes, biosynthesis of vancomycin group antibiotics, glycan degradation, lipopolysaccharide biosynthesis, lipoic acid metabolism, and vitamin B_6_ metabolism. In contrast, BA supplementation increased (LDA > 2, *P* < 0.05) the relative abundances of microbial genes involved in the synthesis and degradation of ketone bodies, *D*-alanine metabolism, cell cycle, PTS, and secondary BA biosynthesis in the IUGR piglets (Fig. 2C). Moreover, dietary BA supplementation to IUGR piglets decreased (LDA > 2, *P* < 0.05) the relative abundances of microbial genes involved in propanoate metabolism and increased (LDA > 2, *P* < 0.05) the relative abundances of microbial genes involved in oxidative phosphorylation and alanine/aspartate/glutamate metabolism when compared with the NBW + BA group (Fig. 2D).

### Effects of BA on the colonic metabolite profiles of weaned piglets with NBW and IUGR

In the positive ion model, PCA indicated a significant separation between the NBW groups with/without BA supplementation (Additional file 5: Fig. S3). Moreover, dietary BA supplementation to NBW and IUGR piglets showed a distinct separation between these two groups. In the negative ion model, PCA indicated a distinct separation between the NBW and IUGR groups and NBW + BA and IUGR + BA groups (Additional file 5: Fig. S3A−H). In addition, OPLS-DA showed distinct separations among the different treatment groups in the positive and negative ion models (Additional file 5: Fig. S3I−P).

A total of 187 differential metabolites were identified, including amino acids (36.89%), lipids (25.13%), carbohydrates (13.37%), nucleotides (9.63%), cofactors and vitamins (6.96%), xenobiotics (6.95%), and other unknown metabolites (1.07%). Sixty-five and 51 metabolites were altered in the IUGR and NBW groups by BA supplementation, respectively, compared to those without BA supplementation. Twenty-two metabolites were common among the four groups.

**Fig. 3:**
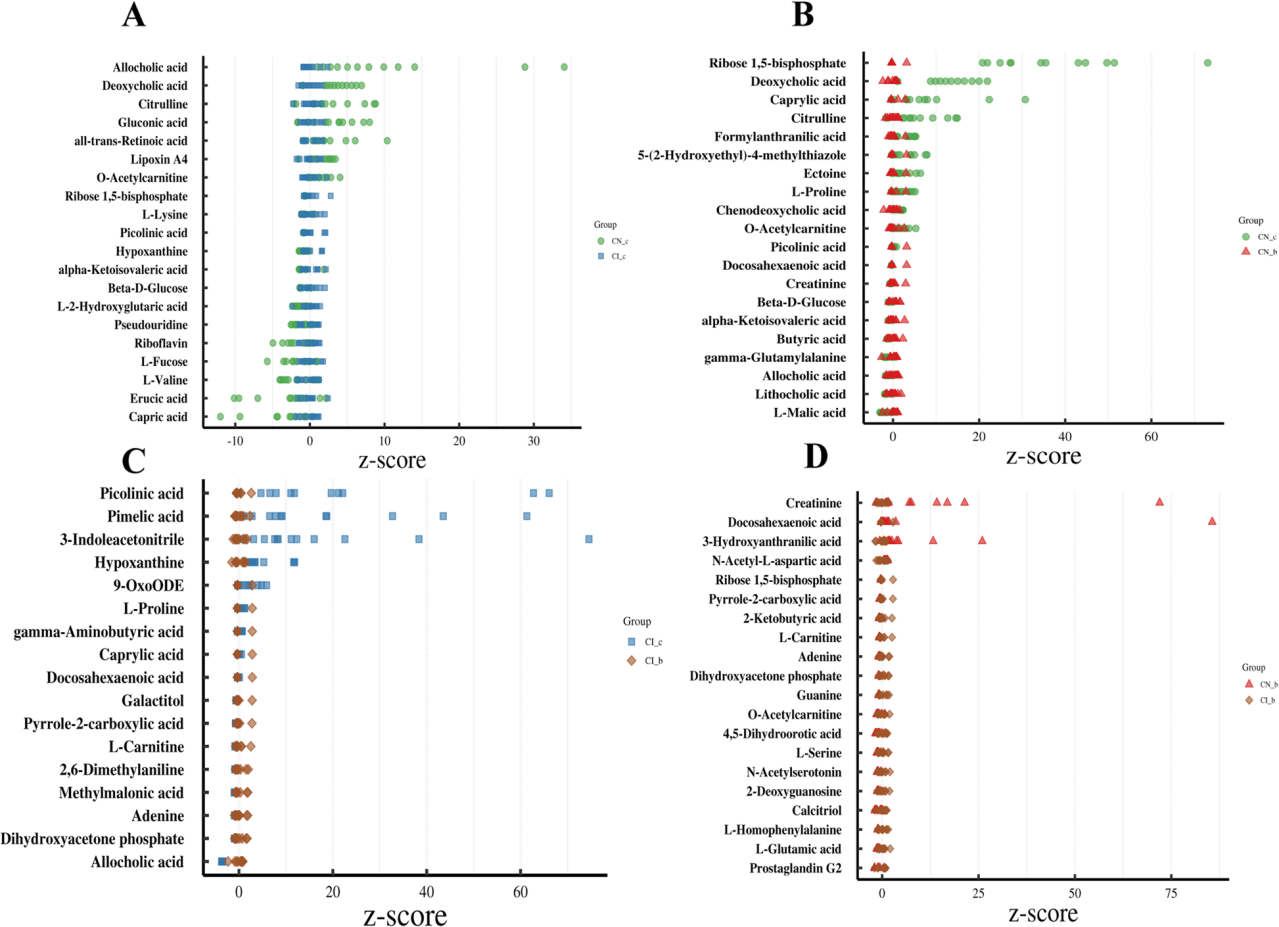
Effects of dietary bile acid (BA) supplementation on differential colonic metabolites in weaned piglets with normal birth weight (NBW) and intrauterine growth retardation (IUGR) (*n* = 11 or 12). **A** represents N_c vs. I_c groups; **B** represents N_c vs. N_b groups; **C** represents I_c vs. I_b groups; **D** represents N_b vs. I_b groups. *N_c* NBW group (NBW piglets fed a basal diet), *I_c* IUGR group (IUGR piglets fed a basal diet), *N_b* NBW + BA group (NBW piglets fed a basal diet supplemented with 400 g/t BA), *I_b* IUGR + BA group (IUGR piglets fed a basal diet supplemented with 400 g/t BA)

As shown in Fig. 3, IUGR piglets had higher colonic concentrations of capric acid, erucic acid, *L*-valine, *L*-fucose, riboflavin, pseudouridine, *L*-2-hydroxyglutaric acid, beta-*D*-glucose, alpha-ketoisovaleric acid, and hypoxanthine and lower concentrations of picolinic acid, *L*-lysine, ribose 1,5-bisphosphate, o-acetylcarnitine, lipoxin A4, all-*trans*-retinoic acid, gluconic acid, citrulline, deoxycholic acid, and allocholic acid, when compared with the NBW piglets (*P* < 0.05) (Fig. 3A). Dietary BA supplementation to NBW piglets increased (*P* < 0.05) the colonic concentrations of *L*-malic acid, lithocholic acid, allocholic acid, gamma-glutamylalanine, and butyric acid and decreased (*P* < 0.05) alpha-ketoisovaleric acid, beta-*D*-glucose, creatinine, docosahexaenoic acid, picolinic acid, o-acetylcarnitine, chenodeoxycholic acid, *L*-proline, ectoine, 5-(2-hydroxyethyl)-4-methylthiazole, formylanthranilic acid, citrulline, caprylic acid, deoxycholic acid, and ribose 1,5-bisphosphate (Fig. 3B).

Dietary BA supplementation to IUGR piglets increased (*P* < 0.05) the colonic allocholic acid, dihydroxyacetone phosphate, adenine, methylmalonic acid, 2,6-dimethylaniline, *L*-carnitine, pyrrole-2-carboxylic acid, galactitol, and docosahexaenoic acid concentrations and decreased (*P* < 0.05) gamma-aminobutyric acid, *L*-proline, 9-oxoODE, hypoxanthine, 3-indole acetonitrile, pimelic acid, picolinic acid, cytosine, nicotine, and 5-(2-hydroxyethyl)-4-methylthiazole concentrations (Fig. 3C). Dietary BA supplementation to IUGR piglets increased (*P* < 0.05) colonic prostaglandin G2, *L*-glutamic acid, *L*-homophenylalanine, calcitriol, 2-deoxyguanosine, N-acetylserotonin, *L*-serine, 4,5-dihydroorotic acid, o-acetylcarnitine, guanine, dihydroxyacetone phosphate, adenine, *L*-carnitine, 2-ketobutyric acid, pyrrole-2-carboxylic acid, and ribose 1,5-bisphosphate concentrations but decreased (*P* < 0.05) the N-acetyl-*L*-aspartic acid, 3-hydroxyanthranilic acid, docosahexaenoic acid, and creatinine concentrations compared to those in the NBW + BA group (Fig. 3D).

### Effects of BA on the colonic metabolism pathways of weaned piglets with NBW and IUGR

**Fig. 4:**
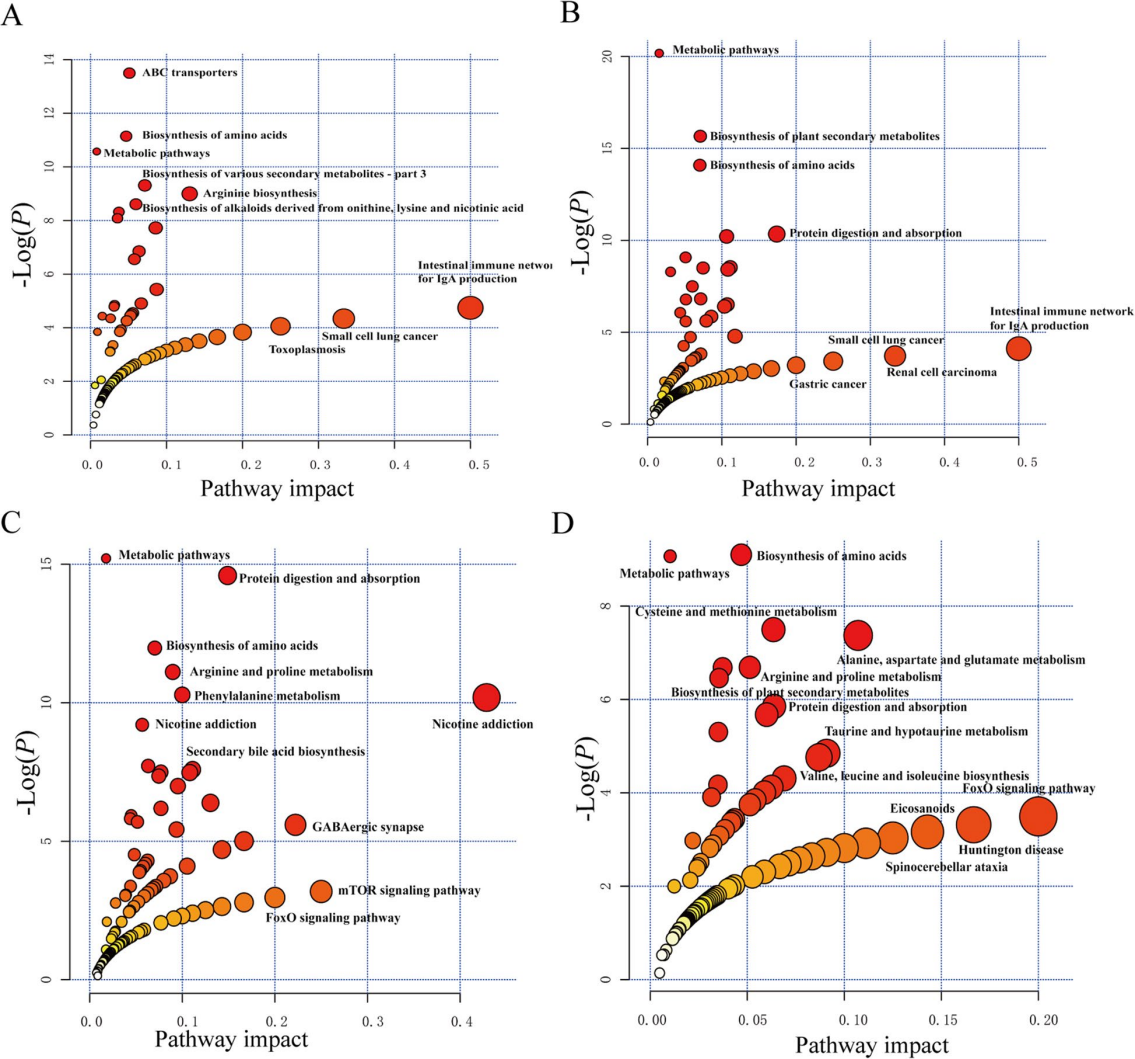
Effects of dietary bile acid (BA) supplementation on colonic metabolic pathways in weaned piglets with normal birth weight (NBW) and intrauterine growth retardation (IUGR) (*n* = 11 or 12). **A** represents N_c vs. I_c groups; **B** represents N_c vs. N_b groups; **C** represents I_c vs. I_b groups; **D** represents N_b vs. I_b groups. *N_c* NBW group (NBW piglets fed a basal diet), *I_c* IUGR group (IUGR piglets fed a basal diet), *N_b* NBW + BA group (NBW piglets fed a basal diet supplemented with 400 g/t BA), *I_b* IUGR + BA group (IUGR piglets fed a basal diet supplemented with 400 g/t BA)

Between NBW and IUGR groups fed a basal diet, differential metabolites with a greater influence on the pathway were mainly enriched in the intestinal immune network for IgA production, small-cell lung cancer, toxoplasmosis, arginine biosynthesis, and gastric cancer (Fig. 4). The differential pathways were enriched in ABC transporters, arginine biosynthesis, pentose phosphate pathway, and aminoacyl-tRNA biosynthesis (Fig. 4A).

Between NBW and NBW + BA groups, differential metabolites with a greater influence on the pathway were mainly enriched in the intestinal immune network for IgA production, renal cell carcinoma, and Th17 cell differentiation. The differential pathways were enriched in protein digestion and absorption, arginine biosynthesis, bile secretion, and pentose phosphate pathway in the NBW piglets with/without BA supplementation (Fig. 4B). Between IUGR and IUGR + BA groups, differential metabolites with a greater influence on the pathway were mainly enriched in the mTOR signalling pathway and GABAergic synapses. The differential pathways were enriched in protein digestion and absorption, arginine and proline metabolism, phenylalanine metabolism, tryptophan metabolism, and bile secretion in the IUGR piglets with/without BA supplementation (Fig. 4C). Between the two BA supplementation groups (NBW + BA and IUGR + BA), differential metabolites with greater influence on the pathway were mainly enriched in the alanine/aspartate/glutamate metabolism, GABAergic synapse, FoxO signaling pathway, and glutamatergic synapse. The differential pathways were enriched in protein digestion and absorption, glycine/serine/threonine, and arginine/proline metabolism in the NBW and IUGR piglets (Fig. 4D).

### Effects of BA on the colonic SCFAs concentrations of weaned piglets with NBW and IUGR

Compared with the NBW piglets, IUGR decreased (*P* < 0.05) the colonic valerate concentration regardless of BA supplementation. Dietary BA supplementation to weaned piglets decreased (*P* < 0.05) colonic butyrate, propionate, and total SCFA concentrations, regardless of IUGR status (Table 2).

### Correlation between colonic metabolites and microbiota of weaned piglets with NBW and IUGR

**Fig. 5:**
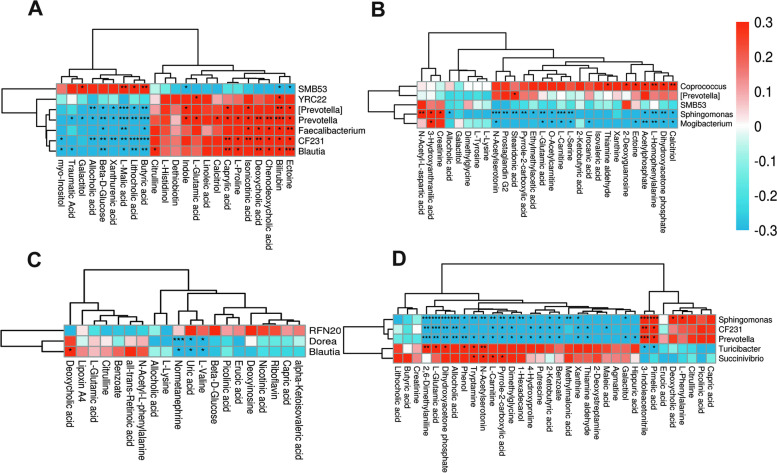
Correlations of colonic differential microbiota abundances and differential metabolite concentrations in weaned piglets with normal birth weight (NBW) and intrauterine growth retardation (IUGR) (*n* = 11 or 12). ^*^P < 0.05, ^**^P < 0.01, and ^***^P < 0.001. The red indicates a positive correlation, and blue indicates a negative correlation. **A**, **B**, **C**, and **D** represent the groups, including N_c vs. N_b, N_b vs. I_b, N_c vs. I_c, and I_c vs. I_b groups, respectively. *N_c* NBW group (NBW piglets fed a basal diet), *I_c* IUGR group (IUGR piglets fed a basal diet), *N_b* NBW + BA group (NBW piglets fed a basal diet supplemented with 400 g/t BA), *I_b* IUGR + BA group (IUGR piglets fed a basal diet supplemented with 400 g/t BA)

The correlations between colonic metabolites and microbiota are shown in Fig. 5. The relative abundance of *SMB53* was positively correlated with butyric acid and *L*-malic acid in the NBW and IUGR groups. The relative abundance of *Prevotella* was positively correlated (*P* < 0.05) with bilirubin and indole, whereas it was negatively correlated (*P* < 0.05) with allocholic acid, butyric acid, and *L*-malic acid. *CF231* was positively correlated (*P* < 0.05) with deoxycholic acid, isonicotinic acid, and caprylic acid while negatively correlated (*P* < 0.05) with butyric acid, beta-*D*-glucose, lithocholic acid, and *L*-malic acid. *Blautia* was positively correlated (*P* < 0.05) with bilirubin, caprylic acid, deoxycholic acid, and ectoine, whereas negatively correlated (*P* < 0.05) with lithocholic acid and butyric acid. *Faecalibacterium* was positively correlated (*P* < 0.05) with ectoine and negatively correlated (*P* < 0.05) with *L*-malic acid. *Prevotella* was positively correlated (*P* < 0.05) with bilirubin, deoxycholic acid, and chenodeoxycholic acid, while negatively correlated (*P* < 0.05) with butyric acid, lithocholic acid, and *L*-malic acid (Fig. 5A). In the NBW and NBW + BA groups, *Coprococcus* was positively correlated (*P* < 0.05) with calcitriol, dihydroxyacetone phosphate, *L*-homophenylalanine, acetylphosphate, ectoine, 2-deoxyguanosine, and thiamine aldehyde, whereas *Prevotella* was positively correlated (*P* < 0.05) with stearidonic acid. *Sphingomonas* was negatively correlated (*P* < 0.05) with dihydroxyacetone phosphate, *L*-serine, *L*-glutamic acid, *L*-carnitine, N-acetylserotonin, N-acetyl-*L*-aspartic acid, pyrrole-2-carboxylic acid, and prostaglandin G2, whereas it was positively correlated (*P* < 0.05) with creatinine, 3-hydroxyanthranilic acid, and N-acetyl-*L*-aspartic acid. *Mogibacterium* was negatively correlated (*P* < 0.05) with calcitriol, acetylphosphate, dihydroxyacetone phosphate, *L*-homophenylalanine, ectoine, urocanic acid, *L*-serine, *L*-carnitine, o-acetylcarnitine, *L*-glutamic acid, and N-acetylserotonin, while positively correlated with 3-hydroxyanthranilic acid (Fig. 5B). In the IUGR and IUGR + BA groups, *Dorea* and *Blautia* were negatively correlated (*P* < 0.05) with normetanephrine, uric acid, and *L*-valine. Moreover, *Blautia* was positively correlated (*P* < 0.05) with deoxycholic acid (Fig. 5C).

In the NBW + BA and IUGR + BA groups, *Sphingomonas* was positively correlated (*P* < 0.05) with 3-indole acetonitrile and pimelic acid, whereas it was negatively correlated (*P* < 0.05) with allocholic acid, dimethylglycine, dihydroxyacetone phosphate, *L*-carnitine, *L*-glutamic acid, N-acetylserotonin, tryptamine, 1-hexadecanol, and 2,6-dimethylaniline. *CF231* was positively correlated (*P* < 0.05) with 3-indole acetonitrile and negatively correlated (*P* < 0.05) with allocholic acid, *L*-glutamic acid, and 2,6-dimethylaniline. *Prevotella* was positively correlated (*P* < 0.05) with 3-indole acetonitrile and negatively correlated (*P* < 0.05) with dihydroxyacetone phosphate, 2,6-dimethylaniline, *L*-glutamic acid, and phenol (Fig. 5D).

**Fig. 6:**
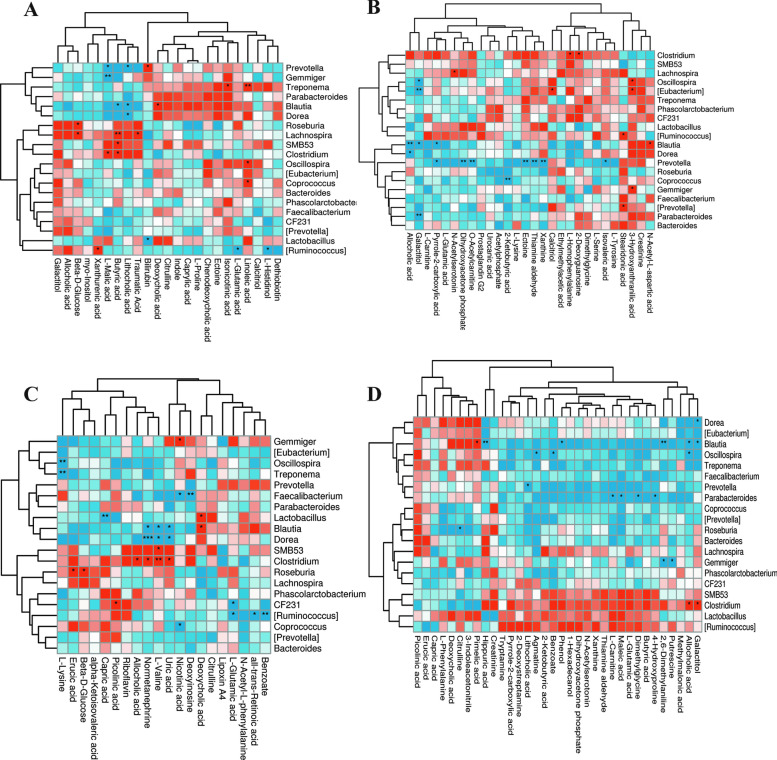
Correlations between microbiota abundances (top 20 genera) and differential metabolite concentrations in weaned piglets with normal birth weight (NBW) and intrauterine growth retardation (IUGR) (*n* = 11 or 12). **A**, **B**, **C**, and **D** represent the groups, including N_c vs. N_b, N_b vs. I_b, N_c vs. I_c, and I_c vs. I_b groups, respectively. ^*^P < 0.05, ^**^P < 0.01. The red indicates a positive correlation, and blue indicates a negative correlation. *N_c* NBW group (NBW piglets fed a basal diet), *I_c* IUGR group (IUGR piglets fed a basal diet), *N_b* NBW + BA group (NBW piglets fed a basal diet supplemented with 400 g/t BA), *I_b* IUGR + BA group (IUGR piglets fed a basal diet supplemented with 400 g/t BA)

Figure 6 shows the correlation between colonic microbiota abundances (top 20 genera) and differential metabolite concentrations. *Lachnospira* was positively correlated (*P* < 0.05) with butyric acid, traumatic acid, and beta-*D*-glucose in the NBW and NBW + BA groups. *Treponema* was positively correlated (*P* < 0.05) with linoleic acid. *Gemmiger* was negatively correlated (*P* < 0.05) with *L*-malic acid in the NBW and NBW + BA groups (Fig. 6A). In the NBW + BA and IUGR + BA groups, *Prevotella* was positively correlated (*P* < 0.05) with dihydroxyacetone phosphate and negatively correlated (*P* < 0.05) with ectoine and thiamine aldehyde (Fig. 6B). In the NBW and IUGR groups, *Oscillospira* was negatively correlated (*P* < 0.05) with *L*-lysine. *Roseburia* was positively correlated (*P* < 0.05) with erucic acid. *Clostridium* was positively correlated (*P* < 0.05) with *L*-valine. *Faecalibacterium* was negatively correlated (*P* < 0.05) with deoxyinosine. *Treponema* was negatively correlated (*P* < 0.05) with *L*-lysine. *Dorea* was negatively correlated (*P* < 0.05) with normetanephrine (Fig. 6C). In the IUGR and IUGR + BA groups, *Dorea* was negatively correlated (*P* < 0.05) with galactitol. *Blautia* was negatively correlated (*P* < 0.05) with allocholic acid, 2,6-dimethylaniline, phenol, galactitol, and hippuric acid while positively (*P* < 0.05) correlated with pimelic acid. *Oscillospira* was negatively correlated (*P* < 0.05) with allocholic acid, benzoate, and agmatine. *Prevotella* was negatively correlated (*P* < 0.05) with lithocholic acid. *Parabacteroides* was negatively correlated (*P* < 0.05) with 4-hydroxyproline, dimethylaniline, malic acid, and *L*-carnitine. *Roseburia* was negatively correlated (*P* < 0.05) with citrulline. *Gemmiger* was negatively correlated (*P* < 0.05) with putrescine and 2,6-dimethylaniline. *Clostridium* was positively correlated (*P* < 0.05) with galactitol and allocholic acid (Fig. 6D).

### Effects of BA on the colonic mucosa inflammation, barrier function, and redox status-related genes expression levels of weaned piglets with NBW and IUGR

Dietary BA supplementation to weaned piglets upregulated (*P* = 0.05) colonic zonula occluden-1 (*ZO-1*) expression and downregulated (*P* < 0.05) Claudin 1 expression regardless of IUGR status. The interaction (*P* < 0.05) between BA and IUGR affected colonic Claudin 1 expression in weaned piglets. Dietary BA supplementation to weaned piglets upregulated (*P* < 0.05) colonic *IL-2*, *IL-6*, *TNF-α*, *IFN-γ*, *CAT*, *GPX*, *Keap1*, *Nrf1*, and *SOD* expressions regardless of IUGR status (Table 3).

**Table 3:** Effects of dietary bile acid (BA) supplementation on colon function-related gene expressions in weaned piglets with NBW and IUGR

Items	NBW		IUGR		SEM	*P*-values		
	** − **BA	** + **BA	** − **BA	** + **BA		IUGR	BA	IUGR × BA
Barrier function-related genes								
Claudin 1	1.00^a^	0.13^b^	0.35^b^	0.34^b^	0.08	0.20	< 0.01	< 0.01
Occludin	1.00	0.97	0.98	1.00	0.06	0.70	0.69	0.87
* ZO-1*	1.00	1.14	1.08	1.29	0.05	0.19	0.05	0.70
* Mucin-1*	1.00	1.10	1.09	1.09	0.05	0.27	0.27	0.32
* Mucin-2*	1.00	1.18	1.14	1.24	0.07	0.52	0.36	0.78
* Mucin-13*	1.00	1.18	1.05	1.06	0.06	0.77	0.45	0.48
Immune-related genes								
* IL-1β*	1.00	0.98	1.41	1.36	0.10	0.06	0.88	0.93
* IL-2*	1.00	1.97	1.17	2.34	0.14	0.08	< 0.01	0.28
* IL-6*	1.00	1.13	0.92	1.35	0.07	0.58	0.04	0.27
* IL-10*	1.00	1.07	0.94	1.24	0.05	0.58	0.06	0.23
* TNF-α*	1.00	1.15	0.88	1.23	0.06	0.83	0.03	0.37
* IFN-γ*	1.00	1.49	1.01	1.73	0.09	0.44	< 0.01	0.48
Antioxidant-related genes								
* CAT*	1.00	1.01	0.73	1.10	0.05	0.33	0.04	0.05
* GPX*	1.00	1.19	0.91	1.42	0.05	0.38	< 0.01	0.05
* Keap1*	1.00	1.38	1.01	1.62	0.06	0.16	< 0.01	0.21
* Nrf1*	1.00	1.08	0.99	1.31	0.04	0.16	0.02	0.16
* Nrf2*	1.00	1.19	1.05	1.32	0.06	0.46	0.06	0.76
* SOD*	1.00	1.11	0.89	1.09	0.03	0.31	0.01	0.48

Values are expressed as means with pooled SEM (*n* = 11 or 12).* P* < 0.05 represents significantly difference. ^a,b^Means with different superscript letters in the same row are significantly different (*P* < 0.05). *CAT* Catalase, *GPX* Glutathione peroxidase, *IL-1β* Interleukin-1β, *IL-2* Interleukin-2, *IL-6* Interleukin-6, *IL-10* Interleukin-10, *IFN*-*γ* Interferon-γ, *Keap1* Kelch-like ECH-associated protein 1, *Nrf1* Nuclear factor erythroid 2-related factor 1, *Nrf2* Nuclear factor erythroid 2-related factor 2, *SOD* Superoxide dismutase, *TNF-α* Tumor necrosis factor-α, *ZO*-*1* Zonula occluden-1. *NBW* Normal birth weight, *IUGR* Intrauterine growth retardation, − *BA* A basal diet without BA, + *BA* A basal diet supplemented with 400 g/t BA

## Discussion

IUGR has been shown to deteriorate intestinal development and function in piglets. Dietary intervention is considered a useful approach for restoring the intestinal health of IUGR in pigs. The present study found that dietary BA supplementation could improve the development and function of the colon, modify the colonic microbiota and metabolic activity, and upregulate colonic antioxidant-related gene expressions in the NBW and IUGR piglets.

The abnormal intestinal function and poor growth performance of IUGR piglets have been widely confirmed [21]. A growing body of evidence showed that the health status and growth performance of IUGR piglets could be improved through nutritional strategies [22]. In the present study, IUGR reduced ADG and final BW of weaned piglets, consistent with previous findings [23]. However, dietary BA supplementation did not accelerate the growth of IUGR piglets. Thus, further studies are needed to investigate the optimal BA supplementation level to facilitate nutrient digestion and promote the growth performance of IUGR piglets.

DAO and *D*-lactate concentrations are usually lower in the plasma of healthy individuals, but significantly increased by the impaired intestinal barrier [24]. Endotoxins produced by Gram-negative bacteria can impair the intestinal barrier and be absorbed into the blood, resulting in systemic or local inflammation [25]. In the present study, IUGR increased plasma and colonic mucosa DAO and *D*-lactate concentrations, which is in accordance with a previous report [26]. Indeed, IUGR can deteriorate hindgut epithelial barrier function in piglets during development [27]. Dietary BA supplementation to NBW and IUGR piglets decreased plasma DAO concentration in the NBW piglets and plasma and colonic *D*-lactate, DAO, and endotoxin concentrations in the IUGR piglets. These findings indicate that dietary BA supplementation may be vital in maintaining colonic barrier function in weaned piglets. In addition, dietary BA supplementation increased the colon length and weight of the NBW piglets, which may be associated with improved colonic function.

The intestinal microbiota participates in various physiological and pathophysiological processes [28] by fermenting undigested food components (mainly fibers and amino acids released from undigested proteins) into SCFAs [29]. Approximately 10% of all nutrients absorbed by an individual originate from intestinal bacterial production [30]. Firmicutes and Bacteroides are considered as the dominant phyla in the intestine of pigs and are associated with energy absorption from nutrient substances and SCFAs production through their fermentative metabolism and carbon degradation properties [31]. In the present study, Firmicutes and Bacteroidetes were the dominant phyla in the colon of weaned piglets with NBW and IUGR, which is in accordance with a previous report [32]. Moreover, dietary BA supplementation increased the abundance of Firmicutes and decreased Bacteroidetes in the weaned piglets, regardless of IUGR status. These results show that dietary BA supplementation altered the colonic microbial community, which might affect the energy metabolism in weaned piglets. However, the effects of these changes on colonic mucosal function require further investigation.

Intestinal dysbiosis can increase the incidence of diarrhea and mortality in animals. In contrast, an increased abundance of beneficial bacteria can enhance immunity in individuals and reduce intestinal diseases. *Lactobacillus* is considered probiotic bacteria, which inhibits pathogenic bacteria reproduction and regulates the balance of intestinal microecology [33]. Moreover, *Lactobacillus* also utilizes fermentative carbohydrates and supplies energy to the intestinal epithelial cells [34]. The present study showed that *Lactobacillus* was the predominant bacteria in the NBW + BA and IUGR + BA groups. These findings indicate that dietary BA supplementation can regulate the colonic microbiota by contributing to the growth of beneficial bacteria.

In biological studies, functional analysis of gut microbiota is generally more informative than the simple composition analysis of different species. In the present study, PICRUSt analysis of colonic microbiota showed that dietary BA supplementation altered several microbial genes functions in the NBW and IUGR piglets, including BA and galactose metabolism. In addition, dietary BA supplementation altered the colonic butanoate, pyruvate, glycolysis gluconeogenesis, propanoate, starch, and sucrose metabolic pathways in the NBW piglets and enhanced lysine biosynthesis, pyruvate metabolism, and DNA replication in the IUGR piglets. These findings indicate that dietary BA has widespread effects on colonic microbial function, including energy and amino acid metabolism. However, further studies are needed to confirm these possible metabolic pathways.

The intestinal microbiota has a complex metabolic activity, not only for its energy and growth but also for producing numerous metabolites in the host tissues [35]. BA derived from hepatic cholesterol can be processed by intestinal microbiota and transformed into secondary BA. For example, intestinal microbiota can convert primary BA into secondary BA through deconjugation and dehydration in the colon [36]. Furthermore, IUGR piglets have impaired intestinal morphology and structure, altered microbial community, and fermentation activity [26]. In the present study, IUGR significantly decreased colonic deoxycholic acid concentration in weaned piglets. Moreover, dietary BA supplementation increased the concentrations of beneficial metabolites such as lithocholic acid, chenodeoxycholic acid, *L*-malic acid, and butyric acid in the NBW piglets. These findings suggest that dietary BA contributed to produce beneficial colonic metabolites in piglets with NBW. Given the abnormal intestinal microbiota of IUGR pigs, the selection of the optimal dosage of BA supplementation requires further investigation.

Furthermore, KEGG database analysis revealed that dietary BA supplementation influences the pathways of bile secretion, protein digestion and absorption, and arginine/proline metabolism in the NBW and IUGR piglets. Previous studies have shown that lithocholic acid is formed through bacterial 7-α-dehydroxylation of primary BA [37]. Lithocholic acid exhibits antibacterial activity and vitamin D receptor modulation [38]. Collectively, these findings suggest that dietary BA has beneficial effects on piglet health by modulating intestinal metabolites. Additionally, dietary BA could affect IgA production and Th17 cell differentiation in the NBW piglets. The mTOR signalling pathway, butanoate metabolism, phenylalanine metabolism, and tryptophan metabolism were modified in the IUGR piglets. These findings suggest that dietary BA can alter the colonic fermentation of nutrients, utilization of amino acids, and innate immunity.

Metabolites derived from the nutrient metabolism by the intestinal microbiota can regulate metabolic activities in the host. Studies have found that linoleic acid is essential to humans and can inhibit inflammation and regulate glucose homeostasis [39]. *Blautia* is widely present in feces, and the intestines of mammals contain *Clostridium* and *Ruminococcus* [40]. Moreover, *Blautia* is significantly correlated with host physiological dysfunctions, such as obesity, diabetes, and inflammatory diseases [41]. The present study showed that dietary BA supplementation did not affect *Clostridium* and *Ruminococcus* abundances in the NBW and IUGR piglets. However, IUGR decreased the *Blautia* abundance in the colon of piglets. In addition, *Blautia* abundance was negatively correlated with lithocholic and butyric acids, while positively correlated with deoxycholic and caprylic acids. These results indicate that decreased colonic *Blautia* abundance in the IUGR piglets may decrease butyric acid concentration, contributing to intestinal damage.

SCFAs are the products of bacterial fermentation of dietary fiber, indigestible oligosaccharides, and some amino acids in the colon, including *Bacteroides*, *Bifidobacterium*, *Fecalibacterium*, and *Enterobacteria*, which can provide various energy sources for intestinal epithelial cells [42]. Research evidence indicates that SCFAs regulate the intestinal microbial community in mammals [43]. The present study showed that IUGR decreased colonic SCFAs contents, which is in accordance with a previous study [44]. Moreover, dietary BA supplementation decreased colonic concentrations of butyrate, propionate, and total SCFAs in weaned pigs. Spearman’s correlation analysis showed that the abundance of *Coprococcus* (producing SCFAs) was positively correlated with colonic acetate, propionate, and butyrate concentrations. These findings indicate that dietary BA supplementation adversely affects SCFA production because SCFAs are an important source of energy production in absorptive colonocytes, and a decrease in the luminal concentrations of these microbial metabolites may be detrimental to the energy status of the colonic epithelium [45]. However, colonic luminal SCFAs concentrations are determined by microbial production and colonic epithelial metabolism [46]. Measuring the capacity of colonocytes to absorb and metabolize butyrate under different experimental conditions would be of major interest.

Tight junction proteins maintain the integrity of the intestinal barrier [47]. IUGR can damage the physical barrier function of the intestinal epithelium in weaned piglets by decreasing the concentrations of intestinal epithelial tight junction proteins [21]. Our results showed that the expression of colonic Claudin 1 was lower in the IUGR piglets. However, dietary BA supplementation upregulated the expression of colonic *ZO-1* in the NBW and IUGR piglets. Collectively, our results suggest that BA plays a crucial role in maintaining the intestinal barrier function.

A recent study confirmed that IUGR impairs immunity by increasing inflammatory factors in weaned piglets [48]. In accordance with a previous study [49], we found that IUGR can increase colonic mucosal pro-inflammatory concentrations (i.e., TNF-α and IL-6) in weaned piglets, while BA supplementation adversely increases the colonic expressions of these two inflammatory cytokines. These effects may be due to the decreased concentration of butyrate in the colonic content induced by BA supplementation, as butyrate exerts anti-inflammatory effects on the colonic mucosa [50].

Intestinal epithelial integrity is closely associated with the redox status of the enterocytes. IUGR can decrease the antioxidant ability of piglets and significantly increase plasma ROS, RNS, and MDA concentrations; conversely, it decreases SOD and GPX activities in the intestinal mucosa [23]. The present study showed that dietary BA supplementation markedly upregulated colonic *CAT*, *GPX*, *SOD*, *Keap1*, *Nrf1*, and *Nrf2* expressions in the NBW and IUGR piglets, especially in the IUGR piglets. Previous studies have indicated that the Nrf2 pathway can effectively regulate intracellular redox status [51]. These findings suggest that dietary BA can improve the intestinal redox status of IUGR piglets via the Nrf2 pathway.

## Conclusions

In summary, dietary BA supplementation improved the development of the colon in the NBW piglets by reducing colonic damage and upregulating colonic antioxidant-related gene expressions in the IUGR piglets. In addition, dietary BA supplementation altered colonic microbiota and metabolites, leading to an increased proportion of beneficial bacteria. However, these beneficial effects must be further balanced with the adverse effects of BA supplementation on the colonic luminal concentrations of SCFA.

## Supplementary Information

**Additional file 1: Table S1. **Ingredients and nutrient levels of the basal diet, % (as-fed basis).

**Additional file 2: Table S2.** Pig specific primer sequences used for RT-PCR.

**Additional file 3: Fig. S1.** Alpha diversity of the colonic microbial community in weaned piglets with normal birth weightand intrauterine growth retardation.

**Additional file 4: Fig. S2.** Effects of dietary bile acidsupplementation on the principal coordinate analysisand partial least square discriminant analysisof the colonic microbial community in weaned piglets with normal birth weightand intrauterine growth retardation.

**Additional file 5: Fig. S3.** The analysis of unsupervised principal component analysisand orthogonal partial least squares discriminant analysisof colonic metabolites in weaned piglets with normal birth weightand intrauterine growth retardation.

## Data Availability

The original contributions presented in the study are included in the article/Supplementary Material, and further inquiries can be directed to the corresponding author.

## Abbreviations

BA: Bile acid
CAT: Catalase
DAO: Diamine oxidase
GPX: Glutathione peroxidase
IUGR: Intrauterine growth retardation
Keap1: Kelch-like ECH-associated protein 1
LEfSe: Linear discriminant analysis effect size
MDA: Malondialdehyde
NBW: Normal birth weight
Nrf1: Nuclear factor erythroid 2-related factor 1
Nrf2: Nuclear factor erythroid 2-related factor 2
OPLS-DA: Orthogonal projections to latent structures-discriminate analysis
PCA: Principal component analysis
ROS: Reactive oxygen species
RNS: Reactive nitrogen species
SCFAs: Short-chain fatty acids
SOD: Superoxide dismutase
