# Supplementary material for: Dietary bile acid supplementation in weaned piglets with intrauterine growth retardation improves colonic microbiota, metabolic activity, and epithelial function
Source: J Anim Sci Biotechnol. 2023 Jul 13;14:99. doi: 10.1186/s40104-023-00897-2 (PMC10339644; doi:10.1186/s40104-023-00897-2)
Supplement: Supplementary file 1 — Additional file 1: Table S1. Ingredients and nutrient levels of the basal diet, % (as-fed basis). [file 40104_2023_897_MOESM1_ESM.docx]

**Table S1** Ingredients and nutrient levels in the basal diet, % (as-fed basis)

| **Items** | **Content** |
| --- | --- |
| Ingredients | |
| Corn | 48.50 |
| Extruded soybean | 12.00 |
| Extruded corn | 10.00 |
| Soybean meal | 7.50 |
| Fermented soybean meal | 5.00 |
| Whey powder | 5.00 |
| Steam fish meal | 3.00 |
| Oil powder | 2.00 |
| Glucose | 2.00 |
| Premix^1^ | 5.00 |
| Total | 100 |
| Nutrient levels^2^ | |
| Digestible energy, MJ/kg | 14.44 |
| Metabolizable energy, MJ/kg | 13.81 |
| Crude protein | 17.50 |
| Crude fat | 4.70 |
| ALys | 1.30 |
| Lys | 1.40 |
| Digestible sulfur-containing amino acids/lysine | 0.55 |
| Digestible threonine/lysine | 0.65 |
| Digestible tryptophan/lysine | 0.19 |

^1^ The premix provided the following per kilogram complete diet: Vitamin A 12,000 IU, Vitamin D_3_ 3,000 IU, α-Tocopherol 50 mg, Vitamin K_3_ 4 mg, Vitamin B_1_ 4 mg, Vitamin B_2_ 10 mg, Vitamin B_6_ 7 mg, Vitamin B_12_ 0.05 mg, Choline chloride 1.00 g, Cu (CuSO_4_ 5H_2_O) 0.50 g, Mn (MnSO_4_ H_2_O) 0.30 g, Zn (ZnSO_4_ H_2_O) 0.30 g, Fe (FeSO_4_ H_2_O) 0.60 g, I (KIO_3_) 10 mg, Se (Na_2_SeO_3_) 1% 10 mg

^2^ Nutrient levels were calculated values
