# Supplementary material for: Dietary bile acid supplementation in weaned piglets with intrauterine growth retardation improves colonic microbiota, metabolic activity, and epithelial function
Source: J Anim Sci Biotechnol. 2023 Jul 13;14:99. doi: 10.1186/s40104-023-00897-2 (PMC10339644; doi:10.1186/s40104-023-00897-2)
Supplement: Supplementary file 2 — Additional file 2: Table S2. Pig specific primer sequences used for RT-PCR. [file 40104_2023_897_MOESM2_ESM.docx]

**Table** **S2** Pig specific primer sequences used for RT-PCR

| **Genes** | **Primers** | **Sequence (5′ to 3′)** | **Size, bp** | **Accession No.** |
| --- | --- | --- | --- | --- |
| CAT | Forward | AGCCTACGTCCTGAGTCTCTGC | 90 | NM_214301.2 |
|  | Reverse | TCCATATCCGTTCATGTGCCTGTG |  |  |
| Claudin1 | Forward | AAGGACAAAACCGTGTGGGA | 102 | NM_001244539.1 |
|  | Reverse | CTCTCCCCACATTCGAGATGATT |  |  |
| GPX | Forward | TGAATGGCGCAAATGCTCAC | 161 | NM_001043534.1 |
|  | Reverse | ATTGCGACACACTGGAGACC |  |  |
| IL-1β | Forward | CCGCCAAGATATAACTGAC | 124 | NM_214055.1 |
|  | Reverse | GCAGCAACCATGTACCAA |  |  |
| IL-2 | Forward | AGCTCTGGAGGGAGTGCTAA | 156 | NM_213861.1 |
|  | Reverse | ACAGCAGTTACTGTCTCATCATCA |  |  |
| IL-6 | Forward | GGATTTCCTGCAGTTCAGCCT | 102 | NM_214399.1 |
|  | Reverse | ACAGGTTTCTGACCAGAGGAG |  |  |
| IL-10 | Forward | ATGGGCGACTTGTTGCTGAC | 154 | NM_001260485.1 |
|  | Reverse | CACAGGGCAGAAATTGATGACA |  |  |
| Keap1 | Forward | CGCCTCATCGAGTTCGCTTACAC | 107 | NM_001114671.1 |
|  | Reverse | GCACGGACCACACTGTCAATCTG |  |  |
| Mucin1 | Forward | GTGCCGACGAAAGAACTG | 152 | XM_040752407.1 |
|  | Reverse | TGCCAGGTTCGAGTAAGAG |  |  |
| Mucin2 | Forward | CTGTGTGGGGCCTGACAA | 157 | XM_021082584.1 |
|  | Reverse | AGTGCTTGCAGTCGAACTCA |  |  |
| Mucin13 | Forward | GCTACAGTGGAGTTGGCTGT | 146 | XM_001234831.7 |
|  | Reverse | GACGAATGCAATCACCAGGC |  |  |
| Nrf1 | Forward | CGATGCTTCAGAATTGCCAACTACAG | 125 | XM_021078993.1 |
|  | Reverse | GCGTTGTCTGGATGGTCATCTCAC |  |  |
| Nrf2 | Forward | CCAATTCAGCCAGCACAACACATC | 149 | XM_003133500 |
|  | Reverse | GACTGAGCCTGGTTAGGAGCAATG |  |  |
| Occludin | Forward | CAGCAGCAGTGGTAACTTGG | 162 | NM_001163647.2 |
|  | Reverse | CAGCAGCAGTGGTAACTTGG |  |  |
| SOD | Forward | GTTGGAGACCTGGGCAATGT | 142 | NM_001123124.1 |
|  | Reverse | CGGCCAATGATGGAATGGTC |  |  |
| TNF-γ | Forward | CCACGCTCTTCTGCCTACTGC | 135 | NM_214022.1 |
|  | Reverse | TCGGCTTTGACATTGGCTACAA |  |  |
| ZO-1 | Forward | GCCATCCACTCCTGCCTAT | 151 | XM_021098856.1 |
|  | Reverse | CGGGACCTGCTCATAACTTC |  |  |
| β-actin | Forward | GATCTGGCACCACACCTTCTACAAC | 107 | XM_021086047.1 |
|  | Reverse | TCATCTTCTCACGGTTGGCTTTGG |  |  |

*CAT* Catalase, *GPX* Glutathione peroxidase, *IL-1β* Interleukin-1β, *IL-2* Interleukin 2, *IL-6* Interleukin 6, *IL-10* Interleukin 10, *IFN*-*γ* Interferon γ, *Keap1* Kelch-like ECH-associated protein 1, *Muc1* Mucin 1, *Muc2* Mucin 2, *Muc13* Mucin 13, *Nrf1* Nuclear factor erythroid 2-related factor 1, *Nrf2* Nuclear factor erythroid 2-related factor 2, *SOD* Superoxide dismutase, *TNF-α* Tumor necrosis factor α, *ZO*-*1* Zonula occluden-1
