# Supplementary material for: Dietary bile acid supplementation in weaned piglets with intrauterine growth retardation improves colonic microbiota, metabolic activity, and epithelial function
Source: J Anim Sci Biotechnol. 2023 Jul 13;14:99. doi: 10.1186/s40104-023-00897-2 (PMC10339644; doi:10.1186/s40104-023-00897-2)
Supplement: Supplementary file 3 — Additional file 3: Fig. S1. Alpha diversity of the colonic microbial community in weaned piglets with normal birth weightand intrauterine growth retardation. [file 40104_2023_897_MOESM3_ESM.docx]

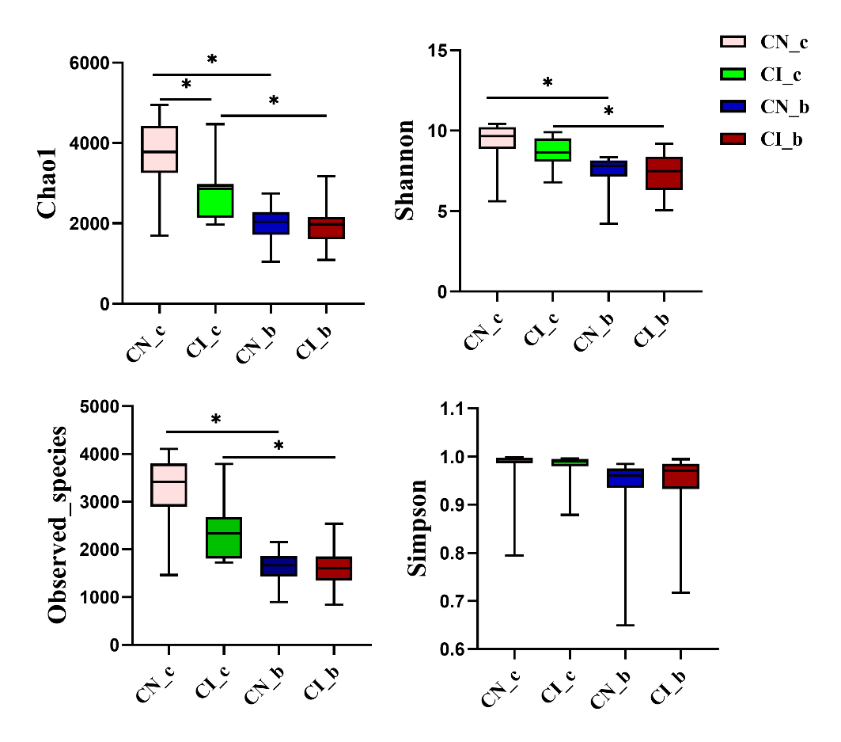


**Fig. S1** Alpha diversity of the colonic microbial community in weaned piglets with normal birth weight (NBW) and intrauterine growth retardation (IUGR) (*n* = 11−12). ^*^*P* < 0.05. *N_c* NBW group (NBW piglets + basal diet), *I_c* IUGR group (IUGR piglets + basal diet), *N_b* NBW + BA group (NBW piglets + basal diet supplemented with 400 g/t BA), *I_b* IUGR + BA group (IUGR piglets+ basal diet supplemented with 400 g/t BA)
