# Supplementary material for: Dietary bile acid supplementation in weaned piglets with intrauterine growth retardation improves colonic microbiota, metabolic activity, and epithelial function
Source: J Anim Sci Biotechnol. 2023 Jul 13;14:99. doi: 10.1186/s40104-023-00897-2 (PMC10339644; doi:10.1186/s40104-023-00897-2)
Supplement: Supplementary file 4 — Additional file 4: Fig. S2. Effects of dietary bile acidsupplementation on the principal coordinate analysisand partial least square discriminant analysisof the colonic microbial community in weaned piglets with normal birth weightand intrauterine growth retardation. [file 40104_2023_897_MOESM4_ESM.docx]

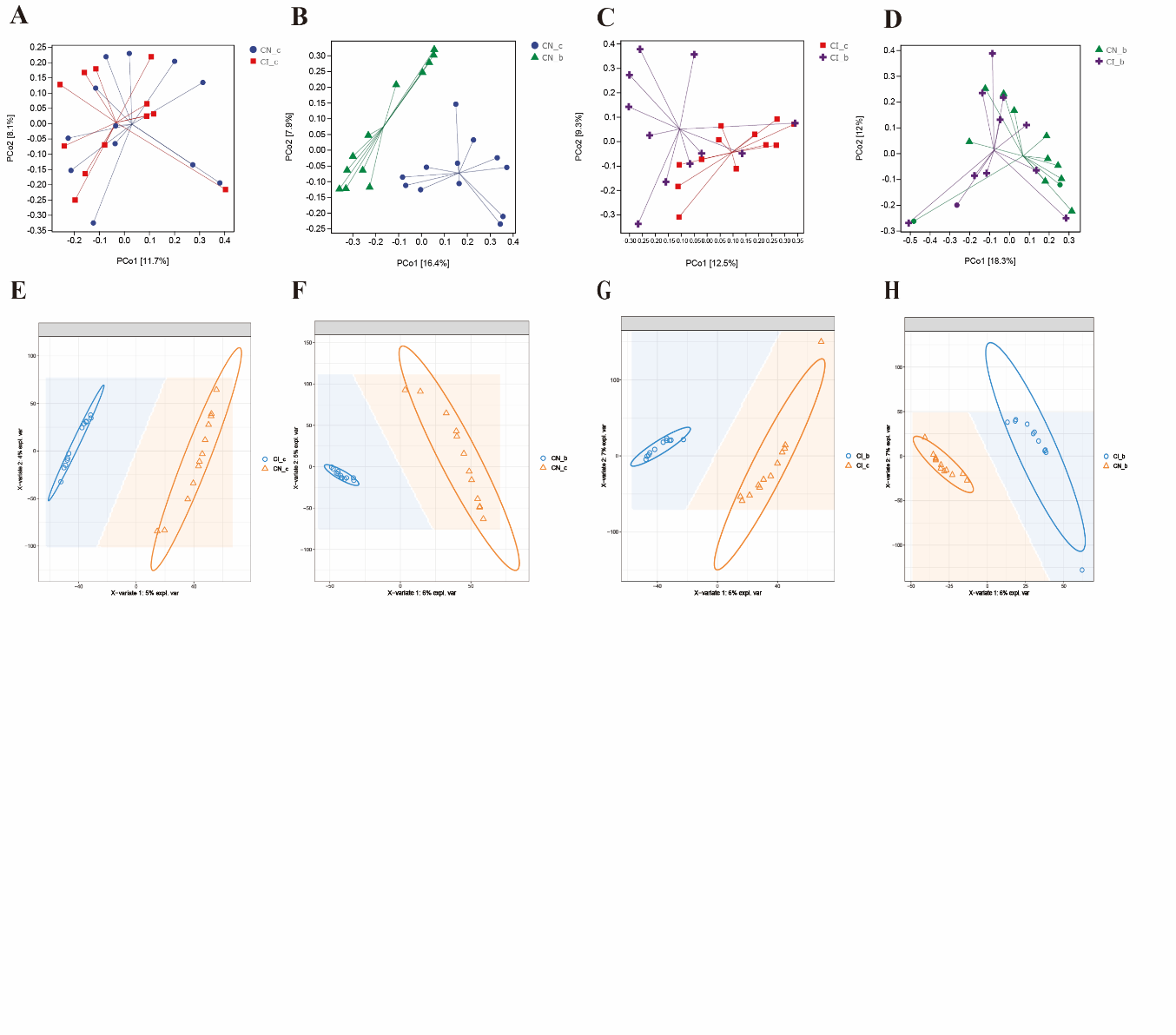


**Fig. S2** Effects of dietary bile acid (BA) supplementation on the principal coordinate analysis (PCoA) and partial least square discriminant analysis (PLS-DA) of the colonic microbial community in weaned piglets with normal birth weight (NBW) and intrauterine growth retardation (IUGR) (*n =* 11−12). **A** and **E** represent N_c vs. I_c groups; **B** and **F** represent N_c vs. N_b groups; **C** and **G** represent I_c vs. I_b groups; **D** and **H** represent N_b vs. I_b groups. *N_c* NBW group (NBW piglets + basal diet), *I_c* IUGR group (IUGR piglets + basal diet), *N_b* NBW + BA group (NBW piglets + basal diet supplemented with 400 g/t BA), *I_b* IUGR + BA group (IUGR piglets + basal diet supplemented with 400 g/t BA)
