# Supplementary material for: Dietary bile acid supplementation in weaned piglets with intrauterine growth retardation improves colonic microbiota, metabolic activity, and epithelial function
Source: J Anim Sci Biotechnol. 2023 Jul 13;14:99. doi: 10.1186/s40104-023-00897-2 (PMC10339644; doi:10.1186/s40104-023-00897-2)
Supplement: Supplementary file 5 — Additional file 5: Fig. S3. The analysis of unsupervised principal component analysisand orthogonal partial least squares discriminant analysisof colonic metabolites in weaned piglets with normal birth weightand intrauterine growth retardation. [file 40104_2023_897_MOESM5_ESM.docx]

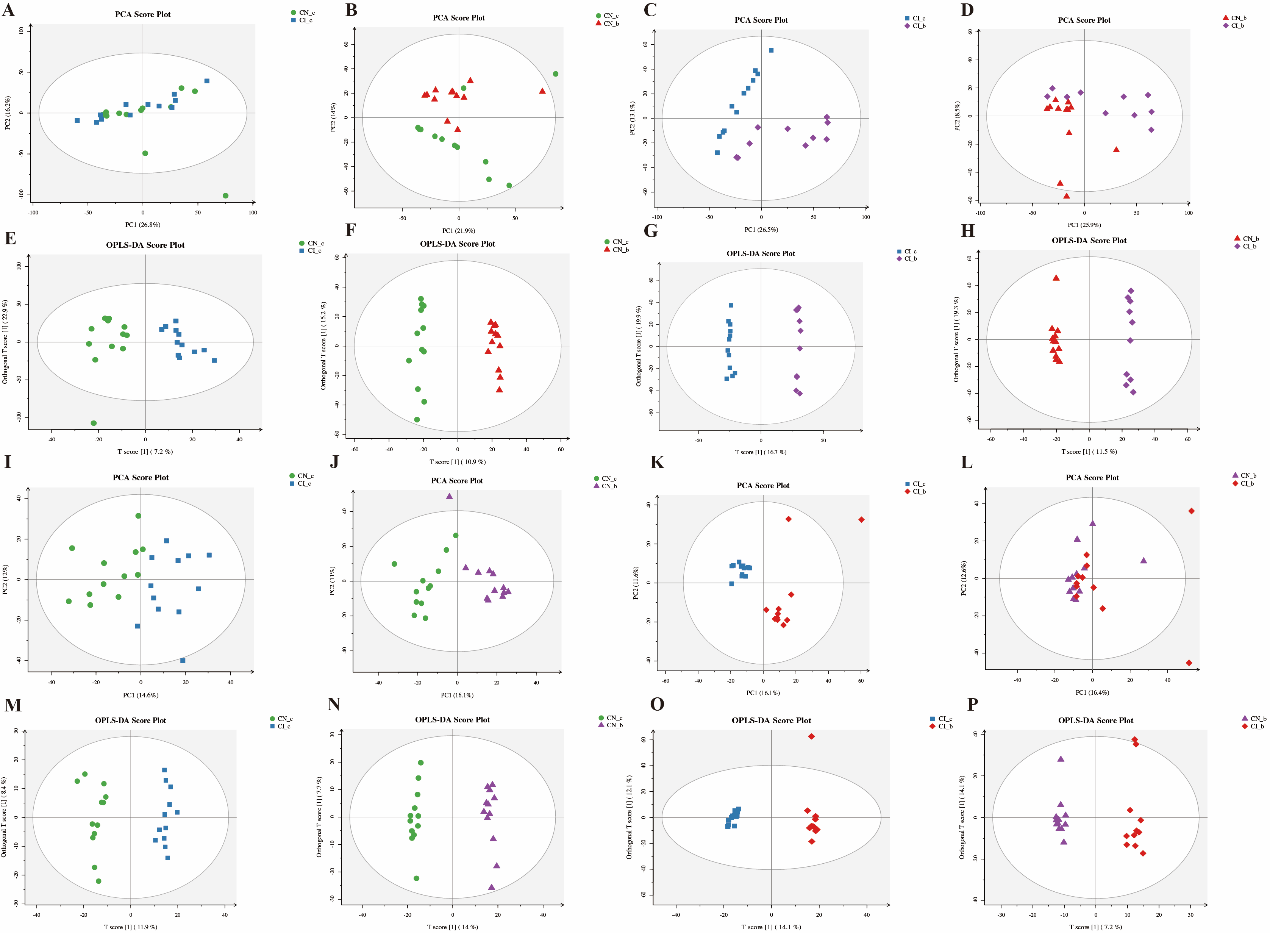


**Fig. S3** The analysis of unsupervised principal component analysis (PCA) and orthogonal partial least squares discriminant analysis (OPLS-DA) of colonic metabolites in weaned piglets with normal birth weight (NBW) and intrauterine growth retardation (IUGR). Each symbol represents one pig (*n* = 11−12). *N_c* NBW group (NBW piglets + basal diet), *I_c* IUGR group (IUGR piglets + basal diet), *N_b* NBW + BA group (NBW piglets + basal diet supplemented with 400 g/t BA), *I_b* IUGR + BA group (IUGR piglets + basal diet supplemented with 400 g/t BA). In the positive ion mode, **A**−**D** represent the PCA plot scores among four groups (N_c vs. I_c, N_c vs. N_b, I_c vs. I_b, and N_b vs. I_b, respectively). **E**−**H** represent the OPLS-DA plot scores among four groups (N_c vs. I_c, N_c vs. N_b, I_c vs. I_b, and N_b vs. I_b, respectively). In the negative ion mode, **I**−**L** represent the PCA plot scores of groups (N_c vs. I_c, N_c vs. N_b, I_c vs. I_b, and N_b vs. I_b, respectively). M, N, O, and P represent OPLS-DA plot scores of groups (N_c vs. I_c, N_c vs. N_b, I_c vs. I_b, and N_b vs. I_b, respectively)
